# 

**Published:** 1871-11

**Authors:** 


					﻿_ PUBLISHED MONTHLY, AT S3 PER ANNUM, IN ADVANCE. i
i.j	-A-TLA-ISTT^	I
QD I	i
I ® :	I
e8 ‘	*
®	Si
°	§
i ®	C	’
{Medical and Surgical JoumLI
<p
•a
■ ~	CD '
§■
S	Si
I!
'2	*0
ik	EDITED BY	’5'
ti	Z3.
i L
I	J. P. LOGAN, M.D.,
I i	» '
'a	■
a	AND	I
' S
W. F. WESTMORELAND, M.D.,	|
rS
a Profetsor of the Principles and Practice of Surgery in Atlanta Medical College, g '
S	B	'
o	o
kO I	c*
. I
i o I	—-----------:----- .
i --I	(Pax et Saientia, sed veritas sin,e timore.
S	  p
;	&
fl	§
' ®	VOLUME IXB-NOVEMBER, 1871,-NUMBER 8.	|
w	«*
a	3
1
O	O
O	  P*
U5
c*
®
1	“
M*
«•	®
2	ATLANTA, GEORGIA:
H
O PLANTATION PUBLISHING COMPANY.
fM
1871.	,
	EACH NUMBER CONTAINS SIXTY-FOUR PACES.
				

